# Safety and efficacy of hematopoietic and mesanchymal stem cell therapy for treatment of T1DM: a systematic review and meta-analysis protocol

**DOI:** 10.1186/s13643-017-0662-9

**Published:** 2018-01-26

**Authors:** Sedigheh Madani, Bagher Larijani, Abbas Ali Keshtkar, Ali Tootee

**Affiliations:** 1 Endocrinology and Metabolism Research Institute, Tehran University of Medical Science, Tehran, Iran; 2 Tehran University of Medical Science, Tehran, Iran

**Keywords:** Diabetes mellitus type 1, Stem cell therapy, Systematic review

## Abstract

**Introduction:**

Insulin standard treatment of T1DM cannot cure the patients as different chronic complications occurred subsequently. Investigations on a curative treatment in T1DM propose cell replacement or maintenance instead of exogenous insulin therapy, but different dimensions of this novel treatment are not clarified.

**Methods and analysis:**

We will include all clinical trials which have evaluated the efficacy MSC or HSC transplantation in T1DM treatment; electronically search bibliographic databases, country registration data banks, and gray literatures; and hand-search two key journals, two experts’ article, and references of the included articles with no language restriction. Primary outcome is the extent of reduction in insulin requirement and secondary outcomes are safety of MSC and HSC therapy, effect of this therapy on diabetic parameters, effect of the rout of transplantation and origin of the MSC or HSC on efficacy of treatment, studies heterogeneity and potential reasons of it. Heterogeneity and its severity will be calculated with Q Cochrane test, *P* value, and *I*^2^ index. STATA software version 12 will be used for meta-analysis. PROSPERO Registration number: CRD42016047176.

**Ethics and dissemination:**

We will publish the systematic review in a peer review journal; as it presents an analysis of published literature, the study does not require ethical approval.

**Strengths and limitations of this study:**

This systematic review and meta-analysis will investigate the efficacy of MSC and HSC transplantation in T1DM treatment with no language restriction. Also we will evaluate gray literatures after hand searching.

This protocol is prepared according to Preferred Reporting Items for Systematic Review and Meta-Analysis Protocols (PRISMA-P).

Two reviewers will evaluate screened full texts, extract data, and asses risk of bias of eligible primary studies independently.

As there is the possibility that we miss some unpublished primary studies due to negative results, we will use funnel plot to detect this and correct it with fill and trim method.

## Introduction

Based on official reports published by International Diabetes Federation, more than 415 million adults had diabetes mellitus in 2015, half of them undiagnosed [1, 2], and, approximately, 542 thousand children suffered from the disease [1]. This heavy burden seriously inflicts health systems in both developed and developing countries.

Although type 1 and type 2 diabetes develop based on different pathologic mechanisms, the consequence is increased blood glucose due to either insufficient secretion of insulin or resistance to the hormone [3]. Management of type one diabetes mellitus (T1DM) which sometimes is referred to as children type or insulin-dependent diabetes necessitates multiple daily insulin injections and pinpricks for measurement of blood glucose levels [3, 4].

### T1DM treatments

As mentioned, the standard treatment of insulin-dependent diabetes is insulin therapy [5]. Insulin therapy needs good training and it is difficult for diabetic children and their families. Moreover, insulin therapy cannot prevent from major diabetes complications which can result in permanent disabilities or even death as a result of hypoglycemia [5]. It is reported that despite insulin therapy, overt nephropathy and severe retinopathy were, respectively, developed in 7 to 30% and 24 to 47% of the T1DM patients in a diabetes clinic of a developed country after 25 years [6, 7].

Since 1999, most innovative treatment approaches for treatment of insulin-dependent diabetes have focused on protect beta cell of pancreatic langerhanc islet against auto immunity or replace these cells with insulin producing cells since 1999 [8, 9]. Islet transplantation with the Edmonton protocol is a choice of beta cell replacement and made 44% of diabetic patients insulin free for at least 3 years according to the Collaborative Islet Transplant Registry (CITR) [8–10]. However, currently, shortage of donors, the cost, and the need for lifelong immune suppression are considered main hurdles to this approach.

Stem cells transplantation (SCT) is a novel treatment for several diseases such diabetes mellitus [11]. In vitro experience was started at 2001–2003 to producing insulin-secreting cells from stem cells [12–14]. Also, different clinical trials investigate mesanchymal stem cells (MSC) from different origins and hematopoietic stem cells (HSC) in T1DM treatment from 2005 [15–18]. There is evidence that MSC transplantation enhanced C peptide level even more than two times and reduced hemoglobin-A1c under 7% in insulin-dependent diabetes and improved chronic complications of diabetes [16, 19]. Also, HSCs showed effective improvement in laboratory parameters in T1DM and T2DM [17, 20, 21]. MSC and HSC therapy have made 20 and 60% of the T1DM patients, respectively, and instances of insulin-free periods for as long as 12 to 24 months are reported [19]. Stem cell therapy is not heir to shortage of supply and is generally considered cost effective.

### Previous literatures

Many primary studies were written in efficacy of the SCT in treatment of T1DM [16–18, 20, 22, 23]. A meta-analysis study has been carried out on clinical efficacy of stem cell therapy in treatment of insulin-dependent diabetes. They analyzed 22 clinical trials in which the effect of multi-potent stem cells were searched in reducing insulin consumption before and after transplantation in type 1 and type 2 diabetic patients. They also showed that MSC and CD34^+^ HSC transplantation are the most effective stem cell in T1DM treatment with 20 to 60% independence to insulin and 7 to 50% insulin dose prominent reduction [19].

### Why we will conduct this systematic review?

The previous meta-analysis studied the efficacy of different kinds of SCT in both T1DM and T2DM treatment until August 2015. This systematic review will stress on nine important points that did not contemplate in El-Badawy' study.

1. Our search will add two important databanks (Scopus, Web of Science), conference papers, and thesis in ProQuest.
2. We will add primary studies that were published between August 2015 and May 2017.
3. Cochrane tool will be used for assessing risk of bias of clinical trials. Also two independent reviewers will perform study selection, primary studies quality assessment, and data extraction. Also we will use consensus to ensure agreement in the conflicts.
4. We define inclusion and exclusion criteria of primary studies.
5. We will evaluate the effect of the primary studies quality on the meta-analysis results.
6. We will evaluate insulin consumption as primary outcome and others as secondary outcomes to perform a classic meta-analysis.
7. Subgroup analysis will be done according to methodological quality and primary studies design.
8. Publication bias, Effect of low sample primary studies, and sensitivity analysis will be considered.
9. We will assess the confidence intervals of the cumulative evidence.

We aim to evaluate the safety (as the lack of prominent adverse events) and efficacy (as the improvement of laboratory parameters for diabetes) of MSC and HSC transplantation for the treatment of T1DM by systematic review of eligible clinical trials, including grey literature, in accordance with the Methodological Standard for the Conduction of new Cochrane Intervention Reviews (MECIR) [24]. We believe that a standard systematic review and meta-analysis of primary clinical studies on stem cell transplantation in T1DM will help clinicians and investigators to design more qualified and effective trials by choosing the best stem cells and the most suitable participants.

### Objective

The objective of this systematic review is to determine the efficacy of MSC and HSM transplantation in treatment of T1DM.

## Method

**Fig. 1:**
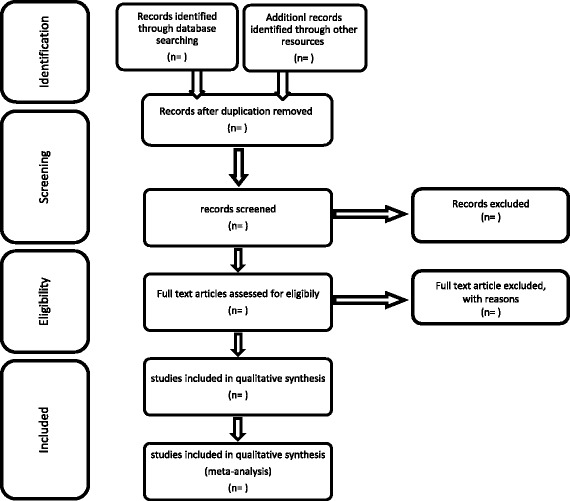
PRISMA flow Diagram for studies screening

This review method merits the PRISMA [25] (Preferred Reporting Items for Systematic Review and Meta-Analysis) checklist guidelines. We will use PRISMA flow diagram to show article number in each step of the search process (Fig. 1). We arranged this protocol according to the PRISMA-P [26] (Preferred Reporting Items for Systematic Review and Meta-Analysis- Protocol) then registered it in the International Prospective Register of Systematic Reviews (PROSPERO; Registration No CRD42016047176; http://www.crd.york.ac.uk/PROSPERO).

### Eligibility criteria

Type of study: We will screen primary clinical trial studies according to the population, intervention, comparisons, and outcome (PICO) criteria.

Primary study inclusion criteria:

- Clinical trials with at least one group of MSC or HSC transplantation on T1DM patients.
- Clinical trials with or without randomization.
- Clinical trials with open-labeled or any degree of blinding method.
- Clinical trials covering all ranges of sample sizes.
- Clinical trials with before and after internal control or a separate control group that receives standard insulin therapy.

Primary studies' Participants:

Inclusion criteria;

- Patients with T1DM (according to the diagnostic criteria of the American Diabetes Association®,(28).
- Patients with any complications.
- Patients in any age group and of any sex.
- Patients with any simultaneous autoimmune disease.

Exclusion criteria:

- Patients with positive human immunodeficiency virus, hepatitis B or C, hematologic malignancies, hematologic or immunodeficiency diseases.

Intervention and comparison:

Inclusion criteria;

- Clinical trials involving the transplantation of MSCs of any origin or any kind of HSCs with or without myeloablation (with cyclophosphamide or any kind of cytotoxic agents),
- Clinical trials involving the transplantations which are applied from any route In which the patients followed up for at least six month and there was at least one follow up after transplantation.

Outcome:

Those studies will be included that have the data for the insulin dose requirement for the enrolled T1DM patients before and after transplantation or the insulin dose requirement of enrolled cases and concurrent controls as a primary outcome.

The secondary outcome inclusion criteria:

Any data about the following parameters will be evaluated;

- Safety of MSC and HSC transplantation in the treatment of T1DM.
- C-peptide levels before and after transplantation or in comparison with controls.
- HbA1c levels before and after transplantation or in comparison with controls.
- Effect of the route of transplantation on the efficacy of the treatment.
- Effect of the origin of the MSCs or HSCs on the efficacy of the treatment.
- Studies heterogeneity and potential reasons for it.

### Search method for finding primary studies

#### Electronic search

First, the author will electronically search primary studies using several common bibliographic databases with no language restriction between 1 January 2000 and 30 September 2016. These databases include PubMed/MEDLINE, SCOPUS, Web of Science (WoS), Embase, Cochrane Central Register of Control Trials (CENTRAL), CINHal, Scientific Electronic Library Online (SCIELO), Indian Citation Index, and Chinese Citation Index.

If at least one study of any country had been fined in electronic search, we will also search that country-specific Clinical Trial Registry system electronically (look like ClinicalTrials.gov, Current Controlled Trials (ISRCTN), EU Clinical Trials Register (EU-CTR), Chinese Clinical Trial Registry, UMIN Clinical Trial Registry (UMIN-CTR), Hong Kong Clinical Trials Register (HKUCTR), Clinical Trials Registry - India, Iran Medex, Iranian Registry of Clinical Trials (IRCT), and Brazilian Clinical Trials Registry (ReBec).

#### PubMed search strategy

Keywords are selected according to synonyms in MeSH database and EMtree database using following combination; “stem cell transplantation”, “hematopoietic stem cell transplantation” and “mesanchymal stem cell transplantation”, “diabetes mellitus type 1” (Appendix 1). We will adopt the PubMed syntax for other databases.

We will create an alert in My NCBI (National Center for Biotechnology Information) to announce us if any new systematic review with basic search strategy is published. If we find more relevant key words during our primary search, we will modify the search syntax.

#### Searching other resources

According to the suggestion of the Institute of Medicine Standards for Systematic Review and the Cochrane Handbook for Systematic Reviews of Interventions [27, 28], we will also include gray literature in this review. We will search Google scholar search engine, conference papers, and thesis (indexed in ProQuest database, SCOPUS, Web of Science) electronically. Two key journals and two experts’ articles according to Scopus report, and references of the included articles will be hand searched.

We will search all searched studies without any language or publication restriction.

#### Search time interval

As mentioned, a meta-analysis was report some clinical trials of stem cell therapy in T1DM and T2DM till august 2015. Since preclinical study of stem cell therapy in T1DM started at 2001–2003 [12–14] and first clinical trials reported in 2005–2007 [15–18], we will study clinical trials between 1 January 2000 and end of September 2016 to cover all probable clinical trials.

#### Study date

The study has been started in November 2016, and we want to end it until august 2017.

### Data collection and analysis

#### Study selection

First, the author will delete the duplications of searched studies by EndNote software version 7, and screen them by title and abstract. Then, two investigators(SM, AT) will independently evaluate screened article eligibility and their methodological quality. Each reviewer will prepare a table that divides articles to included, excluded, or border line and determine articles’ methodological quality. Finally, two reviewers will discuss and agree about their tables differences. Eligible articles will be selected after authors’ consensus. Two reviewers agreement will report with к index.

#### Data extraction

Data extraction form will be designed after reading five primary studies as pilot. Two investigators independently will extract data from final eligible articles by using designed data extraction form and match the forms with consensus.

Extracted data will include following items:

1. Baseline characteristic of primary study: count of study arms, degree of blindness, kind of allocation, study design, study quality score, title, journal, first author name, publication date, trial performing location, starting and finishing date of the trial, sample size, and follow-up duration.
2. Participant characteristics: gender, age, time from T1DM diagnosis, HLA typing, positive interleukins,positive auto-antibody history, and diabetic ketoacidosis history.
3. Intervention and comparison data: kind of transplanted stem cell, route of transplantation, number of transplanted cells per kilogram body weight, number of each treatment group, follow-up visit interval, Randomization, blinding, withdrawal,
4. Outcomes measures: distribution of insulin requirement dose as primary key measure. Any adverse event (as minor, intermediate, and life threatening), C peptide level, HbA1c level, fasting blood sugar, postprandial blood glucose, and auto antibody level. These items will be extracted before transplantation and in each follow-up visit in each treatment group of eligible clinical trials.

#### Data management in specific condition

If any article seems duplicated, we will contact with corresponding author and include the more valid version if necessary. For managing missing data, we will contact with corresponding author three times and we will delete the study if we do not receive any reply. In loss to follow up and different follow-up period of studies, we will conclude similar follow-up period of the studies to minimize the missing data.

#### Heterogeneity assessment

Based on Cochrane Handbook for Systematic Reviews of Interventions [27], we will use *Q* Cochrane test and its *P* value to evaluate heterogeneity between primary studies and *I*^2^ statistic for assessing heterogeneity severity. We will evaluate the severity of heterogeneity before performing a pooled analysis.

We will assess some factors that can affect the heterogeneity such as: primary study quality score and design, kind of stem cell, history of diabetic keto acidosis and auto antibodies, time from diagnosis, and intervention [19]. We will also use statistical heterogeneity results in addition to our decision about the effect size of included trials on meta-analysis results; then, we will choose random effect model or fixed effect model for meta-analysis [29]. A *P* value less than 0.05 will be considered as statistically significant.

#### Publication bias assessment

We will assess publication bias by funnel plot, Egger’s plot or test, and Begg’s plot and tests (Egger’s plot for ten or less primary trials, and funnel, Egger’s, and Begg’s plots for more). If publication bias is not ignorable, we will use fill and trim method to correcting the probable publication bias.

### Risk of bias in primary studies

Two investigators will independently assess the selected articles using Cochrane guidelines for evaluation of clinical trials [30] and evaluate their methodological quality. We will modify Cochrane tool for clinical trials and add two questions after considering Delfi list [31];

1. Has the clinical trial concurrent control group?
2. Are the treatment groups similar at the most important prognostic indicators?

Each author will score articles’ methodological quality. Finally reviewers will discuss and decide about the score differences. Final scores will be selected following reviewer’s consensus.

### Data synthesis

#### Descriptive data

Two reviewers will independently extract data in two separate tables for each eligible trial. A table will consist of methodological quality assessment and the other will contain study characteristic, study duration, participant baseline characteristics, important prognostic factors history in each treatment group, sample size, kind of stem cell, and the route of transplantation, follow-up period, the baseline and each visit means of laboratory parameters of diabetes with their standard deviations in each treatment group, number of insulin-free patient and the duration, and adverse events. Data synthesis will be performed on key measures with standardized mean difference, and its 95% confidence interval in quantitative outcomes and with risk ration and its 95% confidence interval in qualitative outcomes. We will anticipate that enough trials exist for performing a meta-analysis for primary and secondary outcomes. We will carry out meta-analysis of the data using STATA software version 12. Based on primary article methodology, we will combine extracted data by using fixed effect model or random effect model and show the results with forest plot chart. We will separately analyze trials with MSC therapy [32, 33], HSC therapy [17, 34–38], or combination therapy [39, 40].

#### Analysis problems

If eligible trials have no standard treatment control groups, first, we will analyze data of follow-up visit with baseline data of intervention group in all trials and then meta-analyze the intervention and control group in subgroup with concurrent control group. If there is any difference in follow-up visit time points, we will choose the most frequent ones for meta-analysis.

#### Subgroup analysis

We will do subgroup analysis for secondary outcomes (c-peptide level, HbA1c level, adverse events, and studies heterogeneity) and effect of transplantation route and type of stem cells on outcomes. If each subgroup has less than four primary trials, we will use meta-regression for analyzing that key measure effect.

#### Sensitivity analysis

We will summarize methodological quality of primary studies question by question in a table according to the Cochrane Tool for Clinical Trials [30] and study effect of any question on the result of our meta-analysis. If the methodological quality has no effect on the analysis results, we will not restrict our analysis to high-quality studies. Also, we will analyze sensitivity of this systematic review results by restriction methods (quality restriction, design restriction, Jack knife method…). On the other condition, we will decide after methodology and endocrinology experts’ consultation.

Finally, we will apply sensitivity analysis to explain the effect of sample size and methodological quality on the robustness of review results.

#### Summary of findings table

We will assess the confidence of cumulative evidence with Grading of Recommendations Assessment, Development and Evaluation (GRADE) approach [41]. We will provide a summary of findings table in five domains (quality assessment by Cochrane tool, *I*^2^ index for heterogeneity, indirectness according to PICO criteria, impression according to CI 95%, and publication bias) for each outcome. Finally, the quality of the cumulative evidence for each outcome will be scored in a table as very low, low, moderate, and high.

#### Ethics and dissemination

We will publish this systematic review and meta-analysis in a peer review journal and may present it in international congresses and events. As this review will not involve human participants, no ethical approval will be sought.

## Discussion

T1DM is one of the most common and problematic chronic diseases in childhood and adolescence in the world [2]. Standard insulin therapy is difficult and has different chronic complications; therefore, finding a new standard treatment seems necessary. Two approaches in T1DM treatment are replacement of destroyed beta cells of pancreas (with total pancreas transplantation [42] or pancreatic islet transplantation [10]) and reduction of auto immunity against beta cells. Whole pancreatic or islets transplantation have difficulties such as lifelong immune suppression and depends on proper human donors. Stem cell therapy with no restriction in primary source is growing in two dimensions, insulin producing stem cells and immune-modulating stem cells. Insulin producing stem cells are most investigated as in vitro and animal studies [43, 44] but different clinical trials studied immune-modulating stem cells such as MSC and HSC in treatment of T1DM patients. Previously, a number of clinical trials on stem cells therapy in T1DM have undergone meta-analysis in a review [19]; however, to our knowledge, there has been no systematic review and meta-analysis on the subject. HSC and MSC seem to be the most effective therapies in making T1DM patients free of insulin consumption [19]. An updated systematic review is needed to study different aspects of MSC and HSC transplantation in T1DM.

This protocol defines our outcomes and method for meta-analysis of the primary trials. The review results will help researchers and clinicians to design better trials and consider important prognostic factors to select potential candidates accordingly. Also, these results will show whether MSC or HSC therapy is more effective and has less adverse effect. We will search all databases and potential sources of gray literature with no language restriction to conduct a better update review and meta-analysis.

## Appendix 1

The details of PubMed syntax will be as mentioned below.

1. Stem, cell
2. Mesenchymal, stromal cell
3. Progenitor, cell
4. Mesenchymal, stem cell transplantation
5. Mesenchymal stem cell, transplantation
6. Precursor, cell
7. hematopoietic, stem cell transplantation
8. hematopoietic stem cell, transplantation
9. stem cell, transplantation
10. 1 or 2 or 3 or 4 or 5 or 6 or 7 or 8 or 9
11. Diabetes mellitus, insulin-dependant
12. Diabetes mellitus, insulin dependant
13. Diabetes mellitus, juvenile onset
14. Diabetes mellitus, juvenile-onset
15. Diabetes mellitus, type 1
16. Diabetes mellitus, sudden onset
17. Diabetes mellitus, sudden-onset
18. Mellitus, sudden onset diabetes
19. Diabetes mellitus, type I
20. IDDM
21. Diabetes, juvenile onset
22. Diabetes, juvenile-onset
23. Diabetes mellitus, brittle
24. Diabetes mellitus, ketosis-prone
25. Diabetes mellitus, ketosis prone
26. Diabetes, autoimmune
27. 11 or 12 or 13 or 14 or 15 or 16 or 17 or 18 or 19 or 20 or 21 or 22 or 23 or 24 or 25 or 26
28. Date of publication: 2000 till 2016/09/30
29. 10 and 27 and 28

(((Cell[tiab] AND Stem[tiab]) OR (mesenchymal[tiab] AND “stromal cell”[tiab]) OR (Cell[tiab] AND Progenitor[tiab]) OR (Transplantation[tiab] AND “Mesenchymal Stem Cell”[tiab]) OR (“Stem Cell Transplantation”[tiab] AND Mesenchymal[tiab]) OR (precursor[tiab] AND cell[tiab]) OR (Transplantation AND “Hematopoietic Stem Cell”) OR (“Stem Cell Transplantation” AND Hematopoietic) OR (Transplantations AND “Stem Cell”))

AND

((“Diabetes Mellitus”[tiab] AND “Insulin-Dependent”[tiab]) OR (“Diabetes Mellitus”[tiab] AND “Insulin Dependent”[tiab]) OR (“Diabetes Mellitus”[tiab] AND “Juvenile-Onset”[tiab]) OR (“Diabetes Mellitus”[tiab] AND “Juvenile Onset”[tiab]) OR (“Type 1” AND “Diabetes Mellitus”) OR (“Diabetes Mellitus” AND “Sudden-Onset”) OR (“Diabetes Mellitus” AND “Sudden Onset”) OR (“Mellitus” AND “Sudden-Onset Diabetes”) OR (“Diabetes Mellitus” AND “Type I”) OR (“IDDM”) OR (“Diabetes” AND “Juvenile-Onset”) OR (“Juvenile Onset” AND “Diabetes”) OR (“Diabetes Mellitus” AND “Brittle”) OR (“Diabetes Mellitus” AND “Ketosis-Prone”) OR (“Diabetes Mellitus” AND “Ketosis Prone”) OR (Diabetes AND Autoimmune)))

AND

2000/01/01:2016/09/30[dp].
